# The Western Corn Rootworm is Strongly Attracted to Floral (*E*)-p-Methoxycinnamaldehyde, but Not or Weakly to Other Compounds of the Oil Pumpkin

**DOI:** 10.1007/s10886-026-01712-8

**Published:** 2026-04-22

**Authors:** Martin Schlager, Stephan Manhalter, Katharina Wechselberger, Zsolt Kárpáti, Stefan Dötterl

**Affiliations:** 1 Department of Environment & Biodiversity, University of Salzburg, Hellbrunnerstrasse 34, Salzburg, 5020 Austria; 2 Audorf 44, Ebensee am Traunsee, 4802 Austria; 3 Österreichische Agentur für Gesundheit und Ernährungssicherheit GmbH, Spargelfeldstrasse 191, Wien, 1220 Austria; 4 Department of Chemical Ecology, Plant Protection Institute, Centre of Agricultural Research, HUN-REN, Budapest, 1116 Hungary

**Keywords:** Floral scent, *Cucurbita*, oil pumpkin, GC-EAD, agricultural pest, *Diabrotica*

## Abstract

The western corn rootworm (WCR, *Diabrotica virgifera virgifera)* is one of the most serious pests in maize. In some parts of Europe, maize is frequently cultivated adjacent to the Styrian oil pumpkin (*Cucurbita pepo pepo* var. *styriaca*). Adult beetles are strongly attracted to the flowers of this cucurbit, likely due to their strong scent, and lay some of their eggs in the soil of the pumpkin crop. This makes crop rotation, an otherwise effective pest control strategy, less effective. Here, we used gas chromatography coupled to electroantennographic detection (GC-EAD) and field biotests to identify floral scent compounds of the oil pumpkin that elicit physiological and behavioural responses in WCR, respectively. We identified 18 non-sex specific EAD-active compounds for WCR. In field tests, 1,4-dimethoxybenzene, the main compound in pumpkin floral scent, was not attractive to WCR, whereas (*E*)-*p*-methoxycinnamaldehyde, a minor compound, was as attractive as the complete pumpkin mixture. This research provides the basis for developing an attract and kill system, to prevent, or at least mitigate, the substantial yield losses caused by WCR.

**Supplementary Information:**

The online version contains supplementary material available at 10.1007/s10886-026-01712-8.

## Introduction

The western corn rootworm (WCR, *Diabrotica virgifera virgifera*) is a major maize pest. This species, called the “billion-dollar beetle”, caused previously yearly damage of about one billion dollars in the US (Bažok et al. 2021). First found near Belgrade, Serbia, in 1992 after arriving from the US, it has since spread throughout most maize-growing areas in Europe (e.g. Bažok et al. 2021). In Europe and throughout the world, crop rotation is an effective means to fight against this pest. In Austria, however, where maize is often rotated with Styrian oil pumpkin (*Cucurbita pepo pepo var. styriaca*), the effectiveness of crop rotation is strongly reduced. This is because WCR beetles are highly attracted to pumpkin flowers, migrating from maize fields to feed on pollen and leaf tissue of pumpkin, and laying up to 30% of their eggs in pumpkin fields (Foltin and Robier 2014). So far, it is unknown how WCR is attracted to the flowers of Styrian oil pumpkin. However, given the strong floral scents of this species (mostly 100 ng -300 ng, and up to 1 µg per flower and hour; Barman et al. 2024) and the finding that the WCR uses volatiles of other pumpkin varieties and species (e.g. *Cucurbita pepo texana*,* C. maxima*) to locate their flowers (e.g. Andersen et Metcalf 1986, Andrews et al. 2007), it is likely that WCR also finds flowers of the Styrian oil pumpkin by their scents. The benzenoid 1,4-dimethoxybenzene is the main compound in the scent of this species, followed by other benzenoids, such as benzyl alcohol and *p*-anisaldehyde. The flowers also release terpenoids, such as linalool and oxides thereof, and nitrogen-bearing compounds, such as phenylacetonitrile, among others (Barman et al. 2024).

Here, we tested flower scent samples of the Styrian oil pumpkin on the antennae of WCR by using gas chromatography-electroantennographic detection (GC-EAD), to identify floral scent compounds that elicit antennal responses in WCR. The physiologically active compounds were then used in field biotests in maize fields to identify attractive compounds. Specifically, we asked: (1) Which chemicals elicit physiological responses in the antennae of WCR, and do both major and minor scent compounds occur among them? (2) Are WCR beetles attracted only or primarily to mixtures of physiologically active compounds, or also to single major or minor compounds?

## Methods and Materials

*Rearing of WCR*. Western corn rootworm beetles (*Diabrotica virgifera virgifera* LeConte) for electrophysiological tests were reared at AGES (Österreichische Agentur für Gesundheit und Ernährungssicherheit GmbH, Vienna, Austria). Field-collected beetles were kept under long-day conditions (16 h/day) at a temperature of 25 °C, with the temperature reduced to 15 °C at night. They were fed young maize leaves, pumpkin slices, and a dough made from invert sugar syrup, brewer’s yeast and cellulose pulp. Containers filled with loose soil were provided for egg-laying and were regularly sprayed with water to prevent them from drying out. The soil containers were replaced every month, and the egg-soil mixture was stored at 4–7 °C afterwards. The duration of the obligatory diapause depends on the origin of the *Diabrotica* population (Krysan et al. 1977), lasting approximately four months for Austrian populations. Once the diapause had finished, the egg-soil mixture was stored at 25 °C. Untreated maize was grown on a floating net in a water bath at 25 °C. After 1–2 weeks, the maize was placed in the breeding boxes with the egg-soil mixture to provide food for the larvae. After 16–25 days at 25 °C, the larvae hatched from the eggs. Fresh maize plants were added to the breeding box as soon as the maize showed signs of wilting due to larval feeding, and this process continued until the adult beetles hatched.

### Scent Sampling and Synthetic Compounds

To obtain samples for electrophysiological measurements (see below), the floral scents of Styrian oil pumpkin (Cucurbita pepo var. styriaca, cultivars Venus, Rustica, Inka, Gleisdorfer, Classic), cultivated at an average of 21 °C at the Botanical Garden of the University of Salzburg, were collected for 2 h via dynamic headspace between 06:00–08:00 using adsorbent tubes filled with 15 mg Tenax-TA (mesh 60–80) and 15 mg Carbotrap B (mesh 20–40, both Supelco, Bellefonte, PA, USA; see also Gfrerer et al. 2022). Tubes loaded with the scents were eluted with 70 µl of acetone (purity 99.5%; see also Table S1), and the samples obtained were stored at -20 °C until used for the measurements. Physiological measurements and behavioural assays (see below) were (also) performed with synthetic compounds (see Table S1).

Scent samples were also collected from flowers (overall 60 samples from the different cultivars) and synthetic mixtures of physiologically active compounds (see below) to determine the total absolute amount and relative amount of these compounds; thus, to being able to obtain synthetic mixtures that match in headspace collections samples of oil pumpkin flowers. Those headspace samples were collected as above; however, the collection time was 4 min only, and volatiles were trapped on smaller adsorbent tubes (length: 2.5 cm, inner diameter: 2 mm) loaded with only 1.5 mg each of Tenax TA and Carbotrap B. Samples were also collected from leaves to being able to identify flower-specific compounds (see also Barman et al. 2024).

### Chemical Analyses

For identification of EAD-active (see below) flower scents of the Styrian oil pumpkin, scent samples were run on a gas chromatograph/mass spectrometer (GC/MS, QP2010 Ultra EI, Shimadzu, Tokyo, Japan), equipped with a MEGA-DEX column (DMT Beta SE, Ø 0.25 mm, film thickness 0.23 μm, length 30 m, MEGA S.r.l., Legnano, Italy). Helium was used as carrier gas at a flow of 3 mL min^-1^, and samples (injection volume: 1 µL) were run with a split ratio of 1:1. Obtained data were analysed using GCMSolution v.4.41 (Shimadzu Corporation, Kyoto, Japan). We tentatively identified components by comparison of their mass spectra and retention indices with data available in the libraries of Adams, FFNSC 2, Wiley9, NIST11, and ESSENTIAL OILS (Hochmuth Scientific Consulting, Hamburg, Germany). The identity of the components was verified by authentic reference standards (see also Table S1).

Samples collected on the small adsorbent tubes were desorbed in a thermal desorption system (TD-20, Shimadzu, Japan) coupled to a GC/MS-QP2010 Ultra (Shimadzu) equipped with a ZB-5 column (Phenomenex, Ø 0.25 mm, film thickness: 0.25 μm, length: 60 m). The desorbed samples were injected into the GC/MS at a split ratio of 1:1 and a constant helium flow of 1.5 ml/min. The GC oven temperature was 40 °C at the beginning and then increased by 6 °C/min until the final temperature of 250 °C, held for 1 min, was reached. The transfer line between GC and MS was heated at 250 °C. The mass spectra were recorded at 70 eV (m/z 34–350). GCMSolution was again used for analysis. The chemical identification of compounds was based on comparison of linear retention indices, which were based on commercially available n-alkanes (C7-C20; Sigma Aldrich, Vienna, Austria), and mass spectra to data available in the libraries of above. The peak areas (TIC) of samples obtained from the scent lures (see below) were compared to those of the flowers.

### Electrophysiological Analyses

Electrophysiological analyses of oil pumpkin floral scent samples and synthetic compounds (Table S1) were performed with a GC-EAD system consisting of a gas chromatograph (Agilent 7890 A, Santa Clara, California, USA) with a flame ionization detector (FID) and an electroantennographic detection (EAD) setup (heated transfer line; 2-channel USB amplifier, IDAC) from Syntech (Kirchzarten, Germany), the same as described by Gfrerer et al. (2022). We injected 1 µl of the samples (injector temperature: 250 °C) at 40 °C oven temperature into the system. The split valve opened (split 1:1) after 30 s, and the oven heated to 220 °C at a rate of 10 °C/min. A MEGA-DEX column (the same as above) was used for the analyses, with the flow rate of the carrier gas (hydrogen) set to 3 ml/min. The column was split into two capillaries at the end by a µ Flow splitter (Gerstel, Mülheim, Germany). One end was connected to the FID (2 m x 0.15 μm), the other to the EAD (1 m x 0.2 μm). To increase gas pressure, 25 ml/min of nitrogen as make-up gas was fed into the split device. The EAD output discharged into a purified and humidified air stream that flowed around the wired beetle antenna. Following anaesthesia with CO2, antennae of both female and male western corn rootworm were cut at the base and tip, and fixed between glass micropipette electrodes (filled with insect Ringer solution: 8.0 g/l NaCl; 0.4 g/l KCl; 0.4 g/l CaCl_2_). Thin silver wires served as electrodes. In total, we took 126 measurements on 36 beetle individuals (21 females, 15 males), of which 92 were used in the analysis. In the other measurements, the antennae were very noisy, making it difficult to discriminate between signals and noise. Compounds eliciting a response in at least 60% of the tested beetles were considered as physiologically active and were identified by GC/MS (see above, and also Gfrerer et al. 2022).

### Behavioral Experiment

To test the attractiveness of EAD-active volatiles, field tests were conducted in summer 2020. In a maize field near St. Pölten, Lower Austria, we installed PAL-sticky sheets (18 cm x 23 cm in the first experiment and 36 cm x 23 cm in the second experiment, csalomontraps.com) commonly used for monitoring of the western corn rootworm, baited with 1 ml of lures (see Fig. S1). The different compounds were diluted in acetone and mixed to match in headspace samples [collected from cotton rolls placed in perforated oven bags to which the samples were added (see below)] their relative amounts as recorded in headspace samples of oil pumpkin flowers (Table S2). The liquid mixtures were applied to acetone-cleaned cotton rolls which were placed in perforated oven bags (Toppits®, approx. 10 × 10 cm) and fixed to the maize plants with thin wires at a height of 1.5 m. The absolute amounts of the compounds released from these lures represented the amounts released by two flowers of the Styrian oil pumpkin. Attracted WCR were caught on the sheets (Fig. S1) and were taken to the lab for evaluation. All lures were tested with 10 replicates.

In the first experiment, we tested a mixture of all compounds (“Pumpkin”: benzyl alcohol, linalool, 2-phenylethanol, phenylacetonitrile, 4-oxoisophorone, 1,4-dimethoxybenzene, (*E*)-linalool oxide pyranoid, *p*-anisaldehyde, (*E*)-cinnamaldehyde, *p*-anisyl alcohol, (*E*)-cinnamyl alcohol, 3,4-dimethoxystyrene, 1,2,4-trimethoxybenzene, benzyl tiglate, (*E*)-*p*-methoxycinnamaldehyde, 4-hydroxy-3-methoxycinnamaldehyde, benzyl benzoate), a mixture with all compounds except 1,4-dimethoxybenzene (“Pumpkin without DMB”), mixtures of biosynthetically and structurally related compounds alone and in combination with compounds of other classes (Terpenes, “Terp”: linalool, 4-oxoisophorone, *(E)*-linalool-oxide pyranoid; Methoxylated benzenoids, “MA”: 1,4-dimethoxybenzene, *p*-anisaldehyde, *p*-anisyl alcohol, 1,2,4-trimethoxybenzene; Non-methoxylated benzenoids, “N_MA”: benzyl alcohol, 2-phenylethanol, benzyl tiglate, benzyl benzoate; Phenylpropanoids, “PhPr”: *(E)*-cinnamaldehyde, *(E)*-cinnamyl-alcohol, *(E)*-p-methoxycinnamaldehyde; N_MA+PhPr: as above; nitrogen-bearing compounds, “N_Sub”: phenylacetonitrile, indole; “Indole-pMOCA”: *(E)*-p-methoxycinnamaldehyde, indole; “Indole-pMOCA+N_MA”: as above; “Indole-pMOCA+PhPr”: as above), and acetone (negative control) for their attractiveness. The “Indole–pMOCA” mixture resembled the commercial product “Klpflor+” (Tóth et al. 2006) in its relative proportions, based on headspace and GC/MS measurements.

In the second set of experiments, we tested the attractiveness of the complete mixture (“Pumpkin”), the individual compounds of the non-methoxylated benzenoids and phenylpropanoids mixtures, which had been behaviorally attractive in the first experiment, and acetone.

For more details about the preparation of the synthetic samples, see Tables S2, S3.

### Statistical Analyses

We analyzed the WCR capture data using a Kruskal–Wallis ANOVA followed by Tukey nonparametric tests to determine whether the lures differed in their attractiveness to the beetles (STATISTICA 7).

## Results and Discussion

**Fig. 1:**
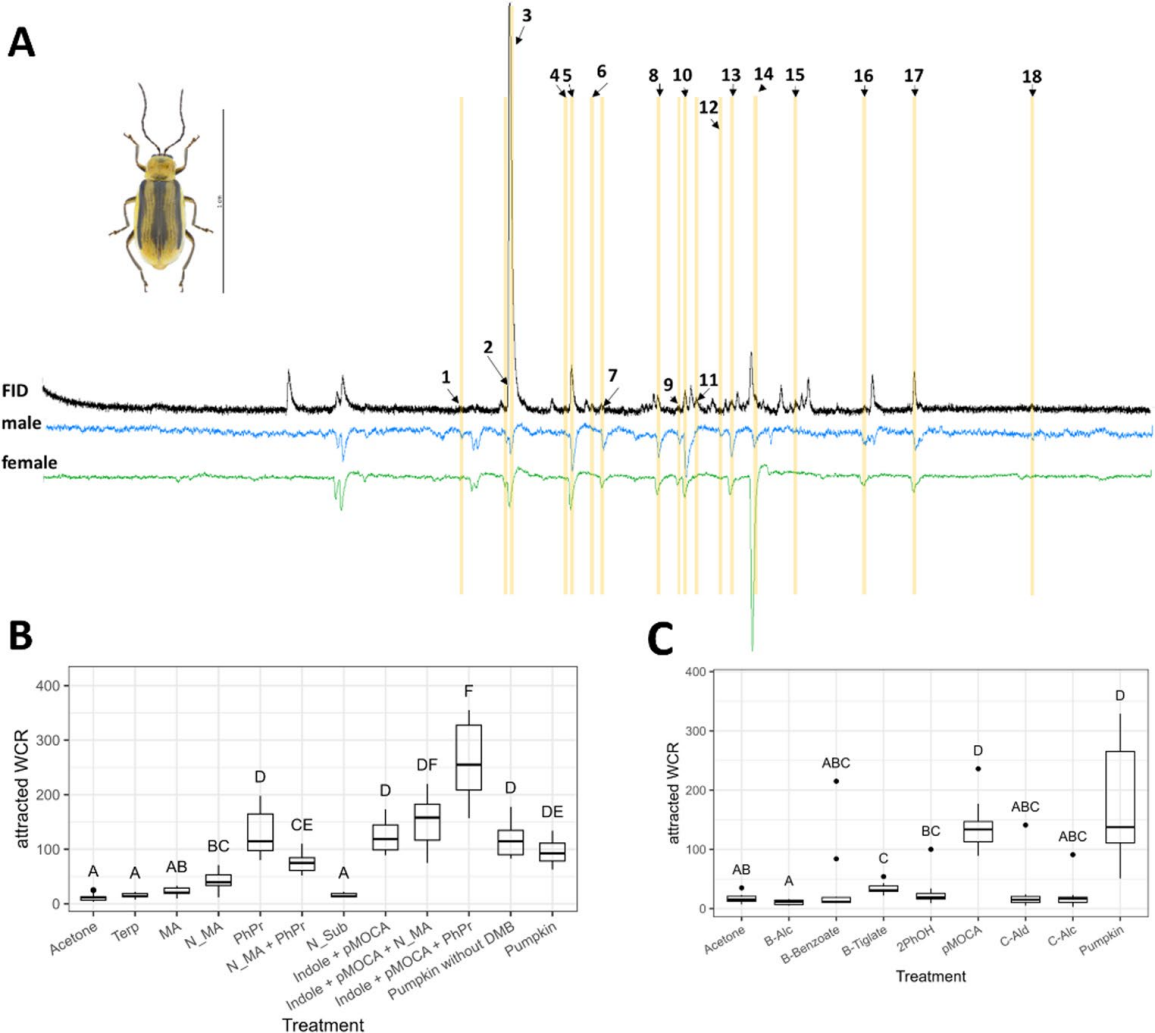
**A**) Examples of coupled gas‑chromatographic and electroantennographic detections (GC‑EAD) of a *Cucurbita pepo var. styriaca* flower scent sample (FID) tested on male and female *Diabrotica virgifera virgifera* antennae. 1: linalool oxide (furanoid)*, 2: linalool*, 3: 1,4‑dimethoxybenzene, 4: (*E*)-linalool oxide (pyranoid), 5: benzyl alcohol, 6: 4‑oxoisophorone, 7: 2‑phenylethanol, 8: 1,2,4‑trimethoxybenzene, 9: phenylacetonitrile, 10: *p*-anisyl alcohol, 11: (*E*)-cinnamaldehyde, 12: 3,4‑dimethoxystyrene, 13: 1‑nitro‑2‑phenylethane, 14: (*E*)-cinnamyl alcohol, 15: benzyl tiglate, 16: (*Z*)-*p*‑methoxycinnamaldehyde, 17: (*E*)-p‑methoxycinnamaldehyde, and 18: benzyl benzoate. *=unknown which of the isomers. Number of WCR (median, quartiles, range without outliers, outliers) attracted in the first (**B**; N = 10602 beetles attracted) and second (**C**, N = 4961 beetles attracted) set of field bioassays.2PhOH: 2-phenylethanol; B-Alc: benzyl alcohol; B-Benzoate: benzyl benzoate; B-Tiglate: benzyl tiglate; C-Alc: (E)-cinnamyl alcohol; C-Ald: (E)-cinnamaldehyde; DMB: 1,4-dimethoxybenzene; MA: methoxylated benzenoids; N_MA: non-methoxylated benzenoids; N_Sub: nitrogen-bearing substances; PhPr: phenylpropanoids; pMOCA: (E)-p-methoxycinnamaldehyde; Pumpkin: complete pumpkin mixture; Terp: Terpenes.Different capital letters indicate significant differences (Kruskal-Wallis ANOVA followed by Tukey nonparametric test). Responses not indicated are either due to contaminations or green leaf volatiles

The GC-EAD recordings on female and male antennae revealed 18 active floral scent compounds (Fig. 1), with females and males responding to the same compounds. These active compounds were mainly terpenoids (e.g., (*E*)-*β*‑ocimene, linalool, 4-oxoisophorone), methoxylated benzenoids (e.g., 1,4‑dimethoxybenzene, 3,4‑dimethoxystyrene, *p*-anisyl alcohol) and phenylpropanoids (e.g., benzenepropanol, (*E*)-cinnamaldehyde, (*E*)-p‑methoxycinnamaldehyde). Some of the active compounds (e.g., benzaldehyde, germacrene D) were not flower-specific but also occurred in leaf scent samples (Fig. 1; data not shown).

In the first set of field tests, all synthetic mixtures except those mixtures containing terpenoids, methoxylated benzenoids, and nitrogen-bearing compounds attracted more WCR than the negative control. Similar numbers of WCR were attracted to the complete pumpkin mixture, the complete pumpkin mixture without 1,4-dimethoxybenzene, “Indole-pMOCA” alone, “Indole-pMOCA” together with non-methoxylated benzenoids, and the phenylpropanoids mixture. The mixture containing “Indole-pMOCA” together with the phenylpropanoids was most attractive, and non-methoxylated benzenoids and non-methoxylated benzenoids together with phenylpropanoids had a weak and intermediate attractiveness, respectively (see also Table S4).

The second set of field tests revealed that most of the non-methoxylated benzenoids and phenylpropanoids were ineffective in attracting WCR, but benzyl tiglate was weakly attractive and (*E*)-*p*-methoxycinnamaldehyde was as attractive as the complete pumpkin mixture (see also Table S5).

Our finding that the complete pumpkin mixture and a mixture that just lacked 1,4-dimethoxybenzene had the same attractiveness to WCR is remarkable, as 1,4-dimethoxybenzene is by far the main compound in the floral scent of the Styrian oil pumpkin (Fig. 1; see also Barman et al. 2024) and known to be attractive to closely related *Diabrotica speciosa* (Ger.) (Ventura et al. 2000). Remarkable is also that a minor component in the floral scent of oil pumpkin, i.e. (*E*)-*p*-methoxycinnamaldehyde was as attractive to WCR as the complete pumpkin mixture (Fig. 1). It attracted both sexes of the WCR, but mainly (70%) females (data not shown). This compound is very rare among floral scents (Knudsen et al. 2006) and was just recently identified for the first time from cucurbit flowers (Styrian oil pumpkin, Barman et al. 2024). Interestingly, however, it has been known as an attractant for WCR and other *Diabrotica* species for three decades (Metcalf and Lampman 1991). Without knowing that (*E*)-*p*-methoxycinnamaldehyde naturally occurs in pumpkin flowers, it has previously been tested on *Diabrotica* spp. as structural analog of pumpkin scents, such as *p*-anisaldehyde and (*E*)-cinnamaldehyde (Metcalf and Lampman 1991).

The only other compound identified as attractive for WCR in the present study was benzyl tiglate. It was a weak attractant and not previously known to be attractive to WCR. As far as we know, benzyl tiglate was even not known to be attractive to any flower visitor before this study, although it is known as a floral scent compound from various plant species and families (Knudsen et al. 2006; Dötterl and Gershenzon 2023).

Overall, we found that (*E*)-*p*-methoxycinnamaldehyde alone is as attractive as a mixture of 18 EAD-active compounds, and is the key component in the floral scent of oil pumpkin responsible for attraction of WCR to its flowers. It is a promising candidate for developing an attract and kill system to fight against WCR in regions where maize is rotated with oil pumpkin. Setting traps at the border of maize fields and adjacent to the pumpkin fields might help to reduce the number of female WCR flying from the maize to the pumpkin field and laying some of their eggs in the soil of the pumpkin fields. Given its rarity among flowering plants (see above), (*E*)-*p*-methoxycinnamaldehyde might not attract large numbers of beneficial insects, such as bees and fly pollinators. Indeed, with the exception of a scatopsid fly (*Swammerdamella brevicornis)*, a very few insects other than WCR were attracted by this compound during our field biotests (data not shown).

## Supplementary Information

Supplementary material.

Supplementary Material 1

Supplementary Material 2

## Data Availability

All data supporting the findings of this study are available within the paper and its Supplementary Information.
