# Correction: The Western Corn Rootworm is Strongly Attracted to Floral (E)-p-Methoxycinnamaldehyde, but not or Weakly to other Compounds of the oil Pumpkin

**DOI:** 10.1007/s10886-026-01723-5

**Published:** 2026-06-11

**Authors:** Martin Schlager, Stephan Manhalter, Katharina Wechselberger, Zsolt Kárpáti, Stefan Dötterl

**Affiliations:** 1 Department of Environment & Biodiversity, University of Salzburg, Hellbrunnerstrasse 34, Salzburg, 5020 Austria; 2 Audorf 44, Ebensee am Traunsee, 4802 Austria; 3 Österreichische Agentur für Gesundheit und Ernährungssicherheit GmbH, Spargelfeldstrasse 191, Wien, 1220 Austria; 4 Department of Chemical Ecology, Plant Protection Institute, Centre of Agricultural Research, HUN-REN, Budapest, 1116 Hungary

**Correction: Journal of Chemical Ecology (2026) 52:37**

10.1007/s10886-026-01712-8.

The published paper “The Western Corn Rootworm is Strongly Attracted to Floral (E)-p-Methoxycinnamaldehyde, but not or Weakly to other Compounds of the oil Pumpkin”, by Martin Schlager, Stephan Manhalter, Katharina Wechselberger, Zsolt Kárpáti and Stefan Dötter needs modification as follows:

1) Please capitalize the title as follows: The Western Corn Rootworm is Strongly Attracted to Floral (E)-p-Methoxycinnamaldehyde, but Not or Weakly to Other Compounds of the Oil Pumpkin

2) 1st sentence of Abstract: “The western corn rootworm…” instead of “The Western corn rootworm…”

3) 1st sentence of Introduction: “The western corn rootworm…” instead of “The Western corn rootworm…”

4) Methods and Materials, Chemical Analyses, 4th sentence: “…their mass spectra and retention indices with data available …” instead of “…their mass spectra with mass spectra available…”.

5) Methods and Materials, Behavioral Experiment, 2nd section: please use “Methoxylated benzenoids” instead of “Methoxylated benzoids”

6) Methods and Materials, Behavioral Experiment, 2nd section: please use “Non-methoxylated benzenoids” instead of “Non-methoxylated benzoids”

7) Methods and Materials, Behavioral Experiment, 3rd section: please use “non-methoxylated benzenoids” instead of “non-methoxylated benzoids”

8) Legend of Figure 1: if possible, please print “A”, “B”, and “C” in bold, i.e. “A) Examples…”; “…attracted in the first (B;…”, and “…and second (C,…”

9) Results and Discussions, section starting with “The only other compounds…”: please use “The only other compound identified as attractive for WCR in the present study was benzyl tiglate. It was a weak attractant and not previously known to be attractive to WCR. As far as we know, benzyl tiglate was even not known to be attractive to any flower visitor before this study, although it is known as a floral scent compound from various plant species and families (Knudsen et al. 2006; Dötterl and Gershenzon 2023).” instead of “The only other compounds identified as attractive forWCR in the present study were benzyl tiglate and 2-phenylethanol. Both compounds were weak attractants and were not previously known to be attractive to WCR before. As far as we know, benzyl tiglate was even not known to be attractive to any flower visitor before this study, although it is known as a floral scent compound from various plant species and families (Knudsen et al. 2006). In contrast, 2-phenylethanol is among the most widespread floral scent compounds (Knudsen et al. 2006) and is known to be attractive to various beneficial flower visitors and repellent to detrimental flower visitors, such as ants (Dötterl and Gershenzon 2023).”

10) Results and Discussions: please delete the last three sections, i.e. “Number of WCR (median, quartiles, range without outliers, outliers) attracted in the first (B; N = 10602 beetles attracted) and second (C, N = 4961 beetles attracted) set of field bioassays. 2PhOH: 2-phenylethanol; B-Alc: benzyl alcohol; B-Benzoate: benzyl benzoate; B Tiglate: benzyl tiglate; C-Alc: (E)-cinnamyl alcohol; C-Ald: (E)-cinnamaldehyde; DMB: 1,4-dimethoxybenzene; MA: methoxylated benzenoids; N_MA: non-methoxylated benzenoids; N_Sub: nitrogenbearing substances; PhPr: phenylpropanoids; pMOCA: (E)-p-methoxycinnamaldehyde; Pumpkin: complete pumpkin mixture; Terp: Terpenes. Different capital letters indicate significant differences (Kruskal-Wallis ANOVA followed by Tukey nonparametric test). Responses not indicated are either due to contaminations or green leaf volatiles.”

The original article has been corrected.

