# Supplementary material for: The Western Corn Rootworm is Strongly Attracted to Floral (E)-p-Methoxycinnamaldehyde, but Not or Weakly to Other Compounds of the Oil Pumpkin
Source: J Chem Ecol. 2026 Apr 22;52(3):37. doi: 10.1007/s10886-026-01712-8 (PMC13102798; doi:10.1007/s10886-026-01712-8)
Supplement: Supplementary file 2 — Supplementary Material 2 [file 10886_2026_1712_MOESM2_ESM.docx]

**SUPPLEMENTARY INFORMATION**

The western corn rootworm is strongly attracted to floral (*E*)-*p*-methoxycinnamaldehyde, but not or weakly to other compounds of the oil pumpkin

Martin Schlager^1,2^, Stephan Manhalter^3^, Katharina Wechselberger^3^, Zsolt Kárpáti^4^, Stefan Dötterl^1,*^

*^1^Department of Environment & Biodiversity, University of Salzburg, Hellbrunnerstrasse 34, 5020 Salzburg, Austria*

^2^*Audorf 44, 4802 Ebensee am Traunsee, Austria*

*^3^Österreichische Agentur für Gesundheit und Ernährungssicherheit GmbH, Spargelfeldstrasse 191, 1120 Wien, Austria*

*^4^Department of Chemical Ecology, Plant Protection Institute, Centre of Agricultural Research, HUN-REN, 1116 Budapest, Hungary*

*^*^ Corresponding author:* [*stefan.doetterl@plus.ac.at*](mailto:stefan.doetterl@plus.ac.at)


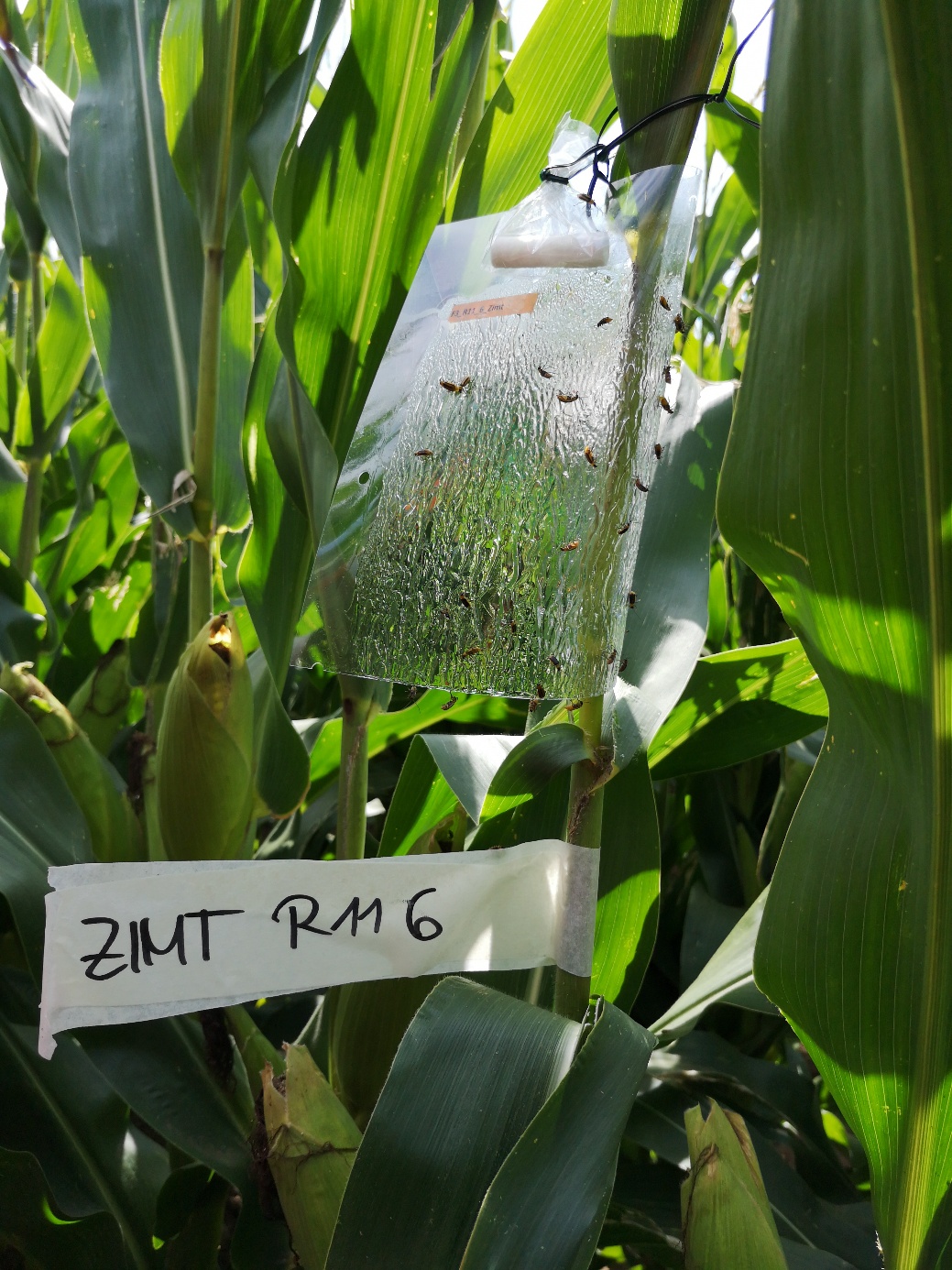


**Fig. S1** Transparent PAL-sticky sheet in a maize field, equipped with a scent lure (cotton roll impregnated with synthetic scent in an perforated oven bag). Several Western corn rootworm beetles were successfully trapped.
